# Narkoseführung mit defektem Cuff

**DOI:** 10.1007/s00101-024-01378-5

**Published:** 2024-01-17

**Authors:** Hans-Peter Reiffen, Anna Elisa auf der Springe, Benjamin Schiller

**Affiliations:** Klinik für Anästhesiologie, Medizinische Hochschule Hannover, Carl-Neuberg-Straße 1, 30625 Hannover, Deutschland

## Anamnese

Ein 42 Jahre alter Patient hatte im Rahmen eines Sturzes multiple Verletzungen erlitten. Eine Unterkieferfraktur war bereits durch Fixierung des Unterkiefers an den Oberkiefer osteosynthetisch versorgt worden; nun stand die unfallchirurgische Versorgung der Extremitätenfrakturen an beiden Armen an. Regionalanästhesiologische Verfahren hatte der Patient abgelehnt.

## Untersuchung

Es besteht keine Mundöffnung; eine Notfallkoniotomie ist anatomisch möglich.

## Diagnostik

In der bronchoskopischen Untersuchung der oberen Luftwege nach Einleitung einer Analgosedierung zeigen sich keine Auffälligkeiten bei allerdings durch vermehrten Speichelfluss eingeschränkter Beurteilbarkeit.

## Therapie und Verlauf

Nach problemloser primär fiberoptischer transnasaler Intubation fällt ein Druckverlust im Cuff auf; es besteht ein erhebliches Luftleck. Daraufhin wird der Tubus problemlos mittels eines Umintubationsstabes gegen ein identisches Modell ausgetauscht. Es zeigt sich dasselbe Problem mit Cuff-Undichtigkeit und Luftleck. Ein dritter Tubus wird zunächst gründlich auf Cuff-Dichtigkeit geprüft und danach in der gleichen Technik platziert. Dieser ist zunächst problemlos zu verwenden; eine Cuff-Beschädigung erscheint unwahrscheinlich.

Intraoperativ zeigt sich erneut ein Luftleck mit einem Leckagevolumen bis zu 200 ml/Atemzug; das Atemminutenvolumen sinkt auf 2,4 l. Eine sichere Beatmung des Patienten ist nicht mehr gegeben.

**Figure 1:**
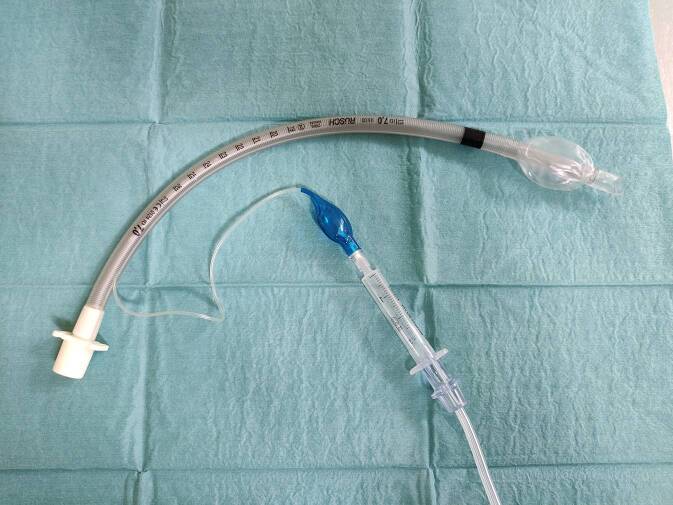

Ein Austausch des Tubus sowie ein Wechsel des Beatmungsverfahrens erscheinen zu diesem Zeitpunkt nicht möglich. Eine weitere Optimierung der Respiratoreinstellungen im Hinblick auf die große Leckage wird als wenig aussichtsreich ebenfalls verworfen. Daraufhin wird der Cuff mittels einer Konstruktion aus einer 2 ml Spritze (B.Braun Injekt® 2ml, Melsungen, Deutschland) und einer Sauerstoffleitung (Intersurgical Oxygen Tube 1,8 m Ref. Nr. 1174000) kontinuierlich nachgeblockt (Abb. 1).

**Figure 2:**
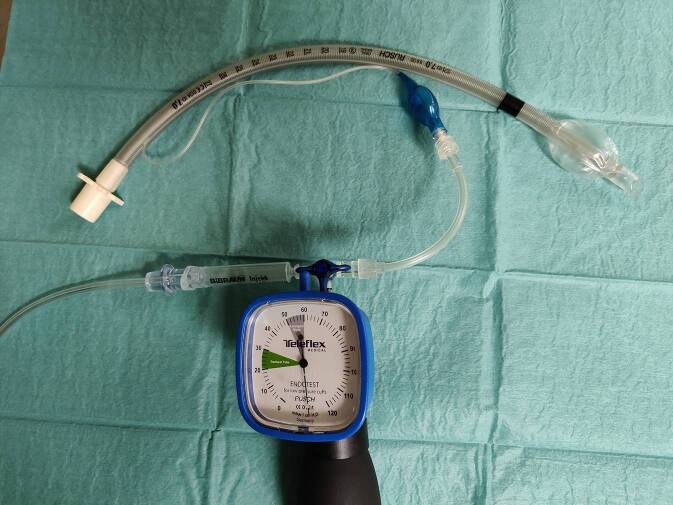

Ante situ war im Vorfeld dieses Konstrukt am ersten verworfenen Tubus geprüft worden, dass ein Flow von 0,5 l/min ausreichend sein dürfte und der Druck distal des Cuff 0 war. Dazu wurde der Tubus in eine 10 ml Spritze gesteckt und anschließend mit der Konstruktion geblockt; die Messung erfolgte an der Spitze; der Konnektor des Tubus war zur Atmosphäre offen. Eine Belastung des Cuff mit Drücken jenseits des inspiratorischen Spitzendrucks erschien unwahrscheinlich, der in der Zuleitung gemessene Druck war allerdings jenseits des Anzeigebereichs des Cuff-Druck-Messers (Abb. 2). Die Möglichkeit zu einem kontinuierlichen Druckmonitoring im Cuff oder einer kontinuierlichen Druckbegrenzung ist nicht vorhanden. Ein Eintauchversuch in eine mit Wasser gefüllte 50 ml Spritze zeigt ein Entweichen der Luft nur nach laryngeal.

Die Narkose kann danach mit einem Luftleck von 0 ml problemlos zu Ende geführt werden.

Der postoperative Verlauf im Aufwachraum und auf der Normalstation gestaltete sich völlig unauffällig. Drei Tage später war eine neuerliche Intubationsnarkose notwendig, und die Inspektion der Trachea zeigte keinerlei Läsionen. Ein Cuff-Leck trat bei dieser zweiten Narkose zu keinem Zeitpunkt auf.

## Diskussion

Der hier gewählte Weg ist eine pragmatische Lösung für ein ansonsten schwer lösbares Dilemma. Selbstverständlich ist sie nicht in jedem Falle anwendbar und wurde hier als Ultima-Ratio-Strategie gewählt. Ganz offensichtlich ist sie nicht mit den rechtlichen Vorgaben an die Patientensicherheit in Einklang zu bringen und somit als Default-Strategie für Cuff-Undichtigkeiten ungeeignet. Dennoch hat sie im vorliegenden Falle eine Beendigung des chirurgischen Eingriffs ermöglicht.

Weiter Untersuchungen am hier beschriebenen Setting, wie z. B. eine Korrelation zwischen dem tatsächlichen Druck im Cuff und der gewählten Flussrate wären im Nachhinein wünschenswert gewesen. Wahrscheinlich sind solche Werte – ebenso wie das Funktionieren der gesamten Konstruktion – aber stark abhängig von der Lokalisation und dem Grad der Beschädigung des Cuff.

## Fazit für die Praxis

Es ist immer gut, einen Plan C zu haben. Diesen präemptiv bei zu erwartenden Problemen zu entwickeln, ist sinnvoll. Die hier beschriebene Lösung kann zu eigenen kreativen Ideen anregen.

